# Asymmetric *N*-Glycosylation in the Tailpiece of Recombinant IgA1

**DOI:** 10.1021/jacs.4c13156

**Published:** 2024-12-06

**Authors:** Manuel David Peris-Díaz, Evolène Deslignière, Shelley Jager, Nadia Mokiem, Arjan Barendregt, Albert Bondt, Albert J. R. Heck

**Affiliations:** † Biomolecular Mass Spectrometry and Proteomics, Bijvoet Center for Biomolecular Research and Utrecht Institute for Pharmaceutical Sciences, Utrecht University, Utrecht 3584 CH, The Netherlands; ‡ Department of Chemical Biology, Faculty of Biotechnology, University of Wrocław, F. Joliot-Curie 14a, Wrocław 50-383, Poland

## Abstract

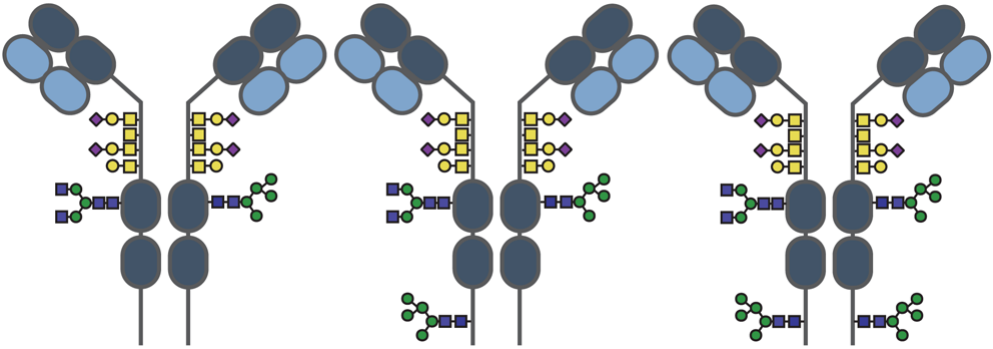

Here, we employed a variety of mass spectrometry (MS)-based approaches, both (glyco)peptide-centric and protein-centric, to resolve the complex glycoproteoform landscape of recombinant IgA1 produced in HEK293 cells. These key immunoglobulins harbor several *N*- and *O*-glycosylation sites, making them considerably more heterogeneous than their IgG counterparts. We provide quantitative data on the occupancy and glycan composition for each IgA1 glycosylation site. Combining all data, we revealed that IgA1 molecules consist of at least three distinct populations with varying *N*-glycosylation site occupancies at the C-terminal tailpiece, namely, one with both glycosylation sites occupied, another with both glycosylation sites unoccupied, and a third asymmetric population with one glycosylation site occupied and the other unoccupied, challenging the prevailing acceptance that IgA1 *N*-glycosylation is symmetrical. This finding is significant, given that the tailpiece is involved in interactions with the J-chain and the Polymeric Immunoglobulin Receptor, and in general as antibody glycosylation is a quality attribute that needs to be carefully monitored, as the presence and nature of these modifications can affect the antibody’s efficacy, lifetime, stability, and binding and/or neutralizing capacities. Optimizing strategies to produce recombinant IgA1 requires efficient and specific quality control analytical strategies, as presented here, which is essential for therapeutic IgA1-based antibody development. We expect that the integrated MS-based strategy presented here may be beneficial to comprehensively characterize the glycoproteoform profiles of IgA1-based therapeutics, thereby improving their production and optimization processes and facilitating the pathway to bring more IgA1-based therapeutics into clinical applications.

## Introduction

Producing biopharmaceuticals of the highest quality in a time- and cost-effective manner is a primary goal in pharmaceutical development and manufacturing. However, many recombinant biotherapeutics, including monoclonal antibodies (mAbs), are complex molecules decorated with post-translational modifications (PTMs) (e.g., glycosylations) that also entail many sequence variations. Antibody glycosylation plays a key role in many processes, such as stability, safety, and affinity for the antigen and Fc receptors.^1^ Due to this, glycosylation is considered a critical quality attribute (CQA) whose comprehensive characterization should be carried out for each batch.^2^

Separation techniques, such as liquid chromatography or capillary electrophoresis, are typically used to identify product variants and impurities in biopharmaceutical products. When coupled with mass spectrometry (MS), they can yield data on several CQAs, by providing additional information about PTMs and genetic or proteolytically induced sequence variants.^3,4^ For mAb glycosylation, the current standards are glycan-centric (glycomics) and glycopeptide-centric (glycoproteomics) profiling. In a glycan-centric analysis, the glycans are released from the mAbs by using enzymes (e.g., PNGases for *N*-glycans), labeled with either 2-AA or 2-AB, whereafter their composition and abundance can be determined by using a variety of separation techniques coupled with optical detection or MS.^4^ This approach works well in the case of IgG-based antibodies, which harbor a single *N*-glycosylation site on each of their heavy chains.^5^ However, the same approach is not applicable for biotherapeutics harboring more than one glycosite because the localization of the glycan is lost when using glycomics. In contrast, a glycopeptide-centric approach, in which glycoproteins are digested into peptides using enzymes such as trypsin, allows to obtain site-specific glycosylation information.^6^ While these peptide-centric methods inform about the mAb’s glycan composition and possibly other PTMs, they often do not provide a full overview of all glycan combinations that sum up to form the glycoproteoform profile of the intact functional and active antibody, which can be derived by protein-centric intact mass measurements.^7,8^

Native MS analyzes molecules in their native-like state, thus preserving the integrity of the antibody.^9^ With sufficient resolving power, such measurements provide a powerful tool to reveal the glycoform compositions in intact antibodies.^10^ Yet, beyond IgG-based mAbs, the heterogeneity of most biopharmaceuticals surpasses the capabilities of native MS to resolve all glycoforms, as many *m*/*z* signals originating from distinct glycoforms can overlap with each other. To alleviate this situation, partial removal of glycans in intact mAbs is commonly employed to reduce the number of closely spaced *m*/*z* signals.^11,12^ However, reducing sample complexity to obtain glycoforms that can be resolved by native MS also results in an incomplete picture of the full glycan heterogeneity, which has implications for the characterization of CQAs. To overcome some of these issues, a tandem MS approach combined with proton-transfer charge reduction was reported and used to disentangle the glycosylation heterogeneity of highly glycosylated biotherapeutics.^13^ Additionally, recent demonstrations have shown that native MS combined with electron-capture charge reduction (ECCR) can resolve highly heterogeneous systems that were not amenable by native MS, such as the SARS-CoV-2 spike protein trimer construct.^14^ By reducing the charge states, the ions are shifted to higher *m*/*z* where the spacing between charge states is larger, enabling the resolution of overlapping peaks.

Immunoglobulin A (IgA) is the most abundantly produced antibody in the human body, specifically at mucosal surfaces and in milk and serum. Most IgA is excreted as a J-chain-coupled dimer onto mucosal surfaces, whereas in serum, IgA is predominantly monomeric. In serum it is the second most abundant immunoglobulin, with concentrations ranging from 1 to 3 mg/mL.^15−17^ In humans, the two IgA subclasses, IgA1 and IgA2, primarily differ in the number of *N*-glycosylation sites, glycan composition, and the fact that only IgA1 contains a 19-amino acid hinge region with nine potential *O*-glycosylation sites. In serum, IgA1 typically represents 90% of total IgA, while in mucosal tissues, IgA1 and IgA2 are more equally distributed.^18^ IgA subclasses have different effector functions: IgA1 seems to be important for immune homeostasis, while IgA2 acts as proinflammatory on neutrophils and macrophages.^17^

Previous MS experiments have reported that the human IgA1 hinge region contains 3–5 core 1 type *O*-glycans in serum.^19^ Furthermore, IgA1 contains two distinct *N*-glycosylation sites per heavy chain: one on the C_H_2 region (N144) and another on the C_H_3 region or, more specifically, on the so-called C-terminal tailpiece (N340). The glycan properties are expected to greatly influence the neutralization capabilities and other functions of IgA.^20^ For example, the glycan on N144 is in a position analogous to that of the glycan on IgG, which has been shown to affect antibody effector functions.^21^ Additionally, the sialic acid residues on the tailpiece *N*-glycosylation site can specifically bind to neuraminidases of virions, interfering with the attachment of viruses that use sialic acids as receptors on the host cell surface.^22^

Thus far, the successful use of antibody-based therapeutics has been dominated by IgG-based mAbs, although recent progress shows that IgA antibodies could represent an exciting avenue for therapeutics, especially as they recruit distinct effector cells.^23−25^ Interestingly, it has been reported that IgA1 exhibited increased *in vitro* SARS-CoV-2 neutralization compared to IgG.^26^ In the treatment of neuroblastoma, the FDA-approved anti-GD2 IgG1 (dinutuximab, ch14.18) improves patient responses.^27^ However, severe pain, partially caused by complement activation in GD2-expressing sensory neurons, is a major dose-limiting side effect of this IgG1. Therefore, an anti-GD2 IgA1 therapeutic variant that does not activate the complement system is currently being explored, thereby reducing the antibody-induced side effects while also enhancing neutrophil-mediated lysis of neuroblastoma cells.^28,29^

Here, we focus on characterizing IgA1-based biotherapeutics. One would *a priori* expect that we can use similar analytical methods as used for IgG1, but the presence of multiple *N*- and *O*-glycosylation sites makes the proteoform landscape of IgA1 far more diverse. To tackle this, we use a variety of hybrid MS approaches, both peptide- and protein-centric, including bottom-up glycoproteomics, native MS, and native ECCR. Only by combining all this data are we able to resolve the (glyco)proteoform landscape of recombinant anti-CD20 IgA1 and recombinant anti-MET IgA1, both produced in HEK293 cells. Remarkably, the data reveal that recombinant IgA1 molecules consist of three populations with different *N*-glycosylation site occupancies on the two heavy chain tailpieces: one population with both tailpiece glycosylation sites fully occupied, another with both tailpiece glycosylation sites unoccupied, and an asymmetric population with one tailpiece glycosylation site occupied and the other unoccupied. These findings are in contrast with the general suggestion that the glycan occupancies on IgA1 are symmetric.

## Results and Discussion

### Site-Specific *N*-Glycosylation Analysis of Recombinant IgA1 by Peptide-Centric Glycoproteomics

To characterize the glycosylation profile of anti-CD20 IgA1 recombinantly expressed in HEK293 cells, we first performed a peptide-centric (bottom-up) glycoproteomics analysis. Each heavy chain of IgA1 has two *N*-glycosylation sites: N144, located on the C_H_2 domain, and N340, located on the tailpiece at the end of the C_H_3 domain. Site N144 was found to be fully occupied with mostly diantennary complex-type glycans (53%) (Figure S1). Other glycans at this site were of the high-mannose-, hybrid-, and extended complex-type glycans (which include bisection and branching) (Figure S1). In contrast, N340 was not always sialylated with an occupancy of around 70%. This site was mostly occupied with high-mannose glycans (33%), followed by extended and diantennary complex-type glycans. The glycosylation profile at N144 aligned well with previously reported data on an anti-HER2 IgA1 produced in HEK293-6E cells.^30^ In contrast, we identified relatively more abundant high-mannose and fewer extended complex-type glycans for N340.

**Figure 1:**
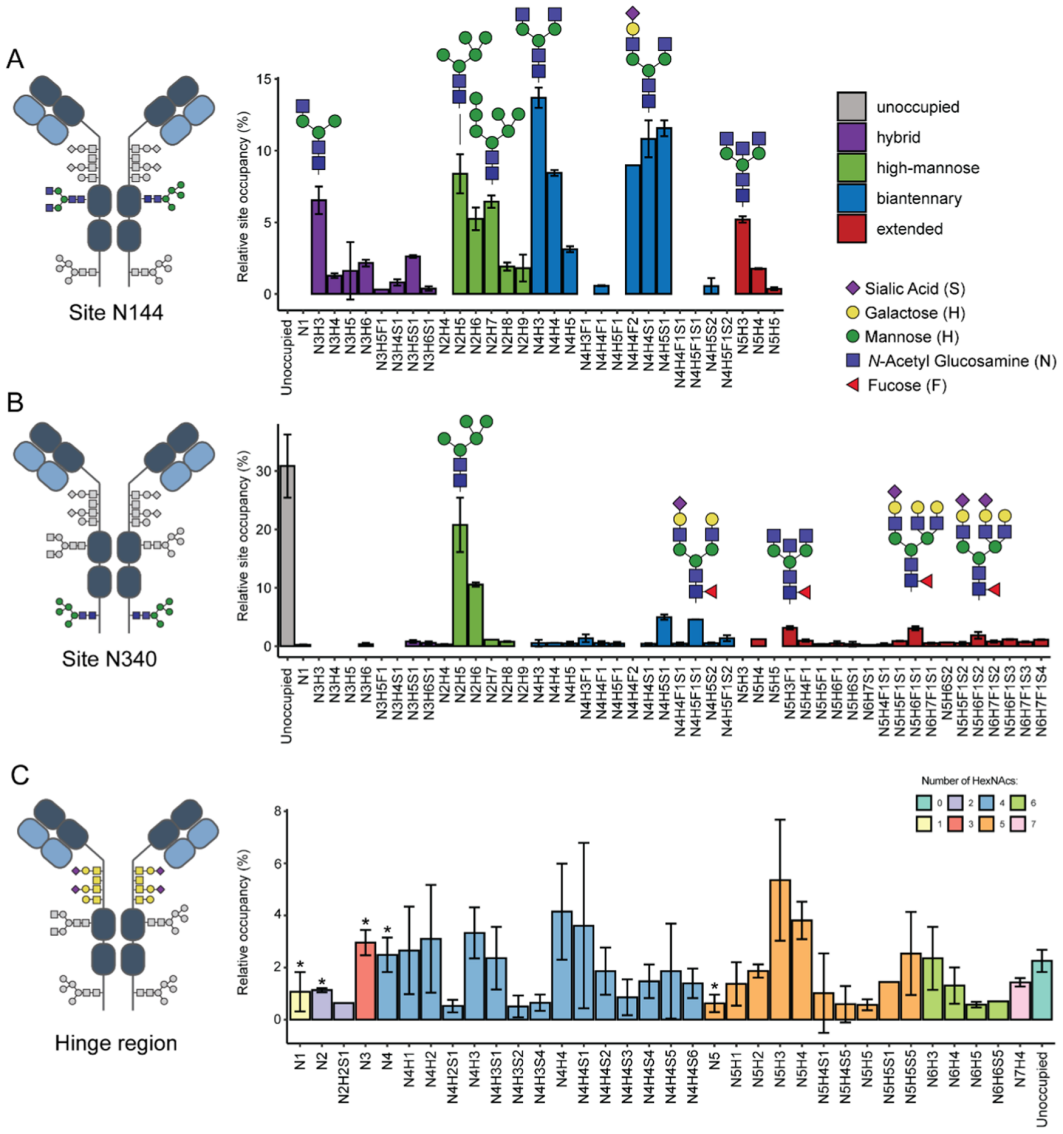
Glycopeptide-centric *N*- and *O*-glycoproteomics of recombinant anti-CD20 IgA1. *N*-glycan compositions and their relative abundance on (A) site N144 and (B) site N340 (across duplicates). Only *N*-glycan compositions with a mean relative intensity higher than 0.1% are shown. The color of the bars represents the glycan characteristic, with green being high-mannose, purple for hybrid, blue for biantennary complex-type, and red for extended complex-type glycans (>4 HexNAc residues). The most common glycans are annotated with their structure. Site N144 is fully occupied, mostly with biantennary complex-type glycans, with low galactosylation and low sialylation, while site N340 is roughly 30% unoccupied (gray bar), and mostly occupied with high-mannose-type glycans and fucosylated complex-type glycans. (C) Cumulative *O*-glycan compositions on the hinge region of recombinant IgA1 and their relative occupancies. Only the *O*-glycans with a mean abundance higher than 0.05% are shown. The error bars in panels (A–C) indicate the standard deviation of the two replicates. Bars without error bar were quantified in only one replicate. Galactose-deficient *O*-glycans are annotated with an asteriks (*).

When individual glycans were examined, the two *N*-glycan sites demonstrated very distinct glycosylation profiles, except for the relatively high abundance of the Man5 glycan (HexNAc_2_Hex_5_ or N2H5) (Figure 1A,B). Site N144 had a very low degree of galactosylation and sialylation, and only 10% of the glycans were fucosylated. Site N340 was more heterogeneous, displaying more, albeit low abundants, glycoforms. These glycoforms were branched, with more galactose moieties, and carried up to four sialic acids. Fucosylated species accounted for 25% at the N340 site.

Apart from *N*-glycosylation, IgA1 harbors a highly *O*-glycosylated hinge region (HR) with nine possible *O*-glycosylation sites. Human serum-derived IgA1 typically has an average of ∼4.5 *O*-glycans per HR, consisting of highly galactosylated and sialylated core 1 type *O*-glycans.^17^ In HEK293 cells, the *O*-glycans on IgA1 were reported to also consist of *N*-acetyl hexosamine (HexNAc or N), hexose (Hex or H), and sialic acid (NeuAc or S) residues, with 4–6 HexNAc residues, 0–5 Hex residues, and 0–4 NeuAc residues.^30^ All of the *O*-glycosylation sites can be studied by focusing on a single tryptic peptide, offering the ability to annotate the total glycan composition of the HR. In the present study, an overwhelming number of more than 150 *O*-glycan compositions of this hinge peptide could be annotated, revealing the intrinsic heterogeneity of this part of the IgA1 molecules. The quantified intensities and total mass increments were matched to the mass deconvoluted spectra of the intact glycopeptides, showing a high overlap (Figure S1B). The most abundant glycan composition found was HexNAc_4_Hex_4_NeuAc_1_ (or N4H4S1), with a relative abundance of just 5% (Figure 1C). Compositions from 1–9 HexNAc residues, 0–9 Hex residues, and 0–13 NeuAc residues could be annotated.

A striking feature from the bottom-up glycoproteomics data was that 30% of the tailpiece glycosite (N340) was seemingly unoccupied. Due to the digestion of the IgA1 molecule into peptides, it is unclear whether this 30% is equally accurate for each of the tailpiece sites or whether this occupancy is asymmetrically alienated. Understanding this stoichiometry provides deeper insight into the assembly of the heavy chains in the full IgA1 antibody and whether this tailpiece glycosylation, or lack thereof, influences assembly. We hypothesized two scenarios: (1) glycosylation influences heavy chain assembly, exclusively generating antibodies that are symmetrically *N*-glycosylated (i.e., 70% of IgA1 molecules are fully glycosylated and 30% are not glycosylated at N340); or (2) assembly is independent of glycosylation, with heavy chains having glycosylated N340 sites assembling with those with unoccupied N340 sites. Since site occupancy at the peptide level does not resolve this question, we next aimed to examine the glycoform landscape on intact IgA1.

### Exposing the Heterogeneous IgA1 Glycosylation by Native Mass Spectrometry

**Figure 2:**
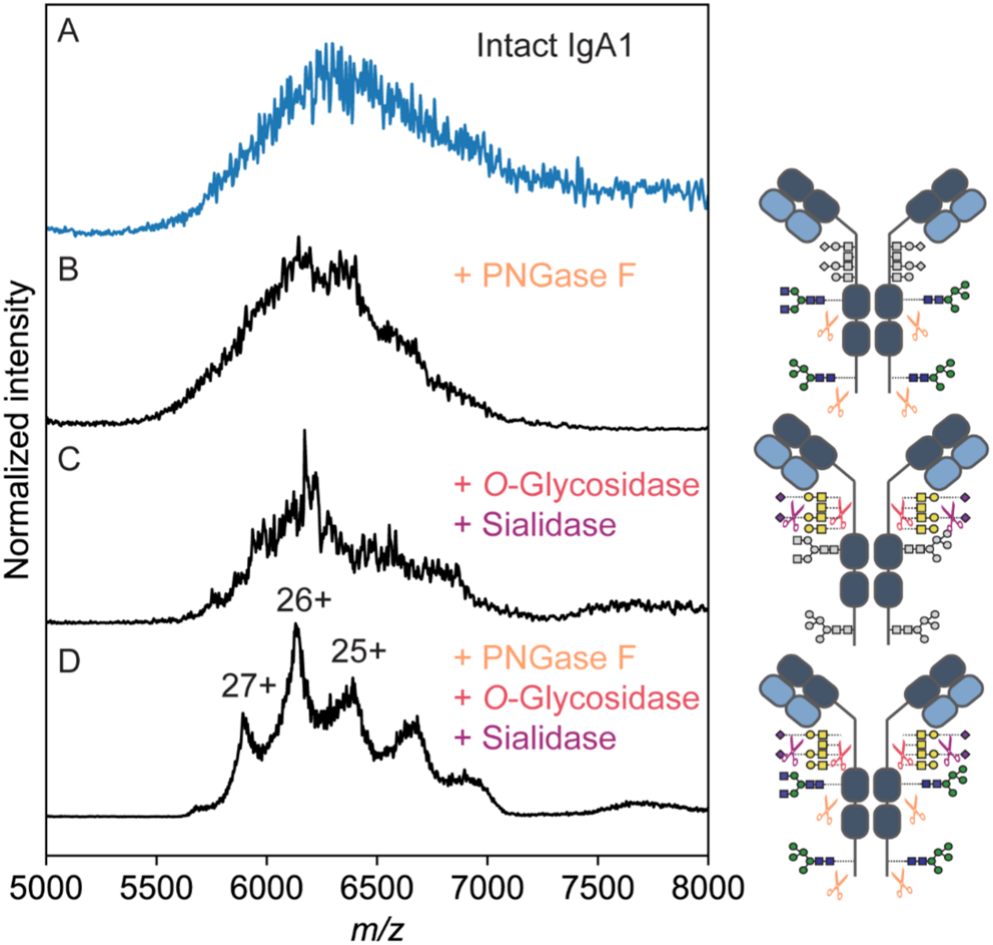
Native MS of recombinant anti-CD20 IgA1 following incubation with different glycosidases. On the right is depicted a schematic illustration of the digestion sites of the used enzymes on IgA1. Native mass spectrum of (A) untreated IgA1, (B) IgA1 treated with PNGase F to cleave off the *N*-glycans, (C) IgA1 digested with *O*-glycosidase to partially cleave off the *O*-glycans, and (D) IgA1 treated with a combination of PNGase F, *O*-glycosidase, and Sialidase targeting *N*-glycans, *O*-glycans, and sialic acids, respectively. The charge states of IgA1 only become partially resolved following incubation by the three glycosidases.

For native MS analysis, recombinant anti-CD20 IgA1 was buffer exchanged into aqueous ammonium acetate and directly infused by static nano-ESI into a Q-Exactive UHMR. Contrary to monoclonal IgG1, the resulting mass spectrum of intact IgA1 was spectrally very dense, with unresolved signals and undistinguishable charge states (Figure S2).^31^ Recording the full glycoproteoform profile of intact IgA1 at increasing transient times to improve the resolution did not lead to better-resolved charge states but instead resulted in a highly congested mass spectrum due to many overlapping ion signals (Figure S2). To understand the respective contributions of *N*- and *O*-glycans to the observed IgA1 heterogeneity, we next performed an enzymatic strategy involving glycosidases. Initially, we sought to reduce the heterogeneity caused by IgA1 *N*-glycans. Removal of *N*-glycans using PNGase F resulted in a less dense mass spectrum, although it still displayed huge heterogeneity (Figure 2A,B). We next considered removing the *O*-glycans using an *O*-glycosidase from *Enterococcus faecalis*, which cleaves core 1 and core 3 *O*-glycans.^32^ Sialidase was also included to remove sialic acids and assist in the cleavage of terminal sialic acids in the *O*-glycans. Reducing the *O*-glycan complexity partially alleviated the heterogeneous ion signals, although it was still insufficient to determine masses (Figure 2C). Incubation of the recombinant IgA1 sample with both PNGase F and *O*-glycosidase to remove *N*- and *O*-glycans resulted in a native mass spectrum where the charge states became resolved (Figure 2D). However, extreme spectral congestion persisted due to numerous overlapping signals in the *m*/*z* space, likely arising from the heterogeneity of the remaining *N*- and *O*-glycans in the IgA1 HR, which could not be resolved by increasing the transient time on the Orbitrap mass analyzer.

### Resolving *N*-Glycoforms on Intact Galactose-Deficient IgA1

#### Native MS Reveals High Heterogeneity of Fc Fragments after OpeRATOR-Induced Cleavage

**Figure 3:**
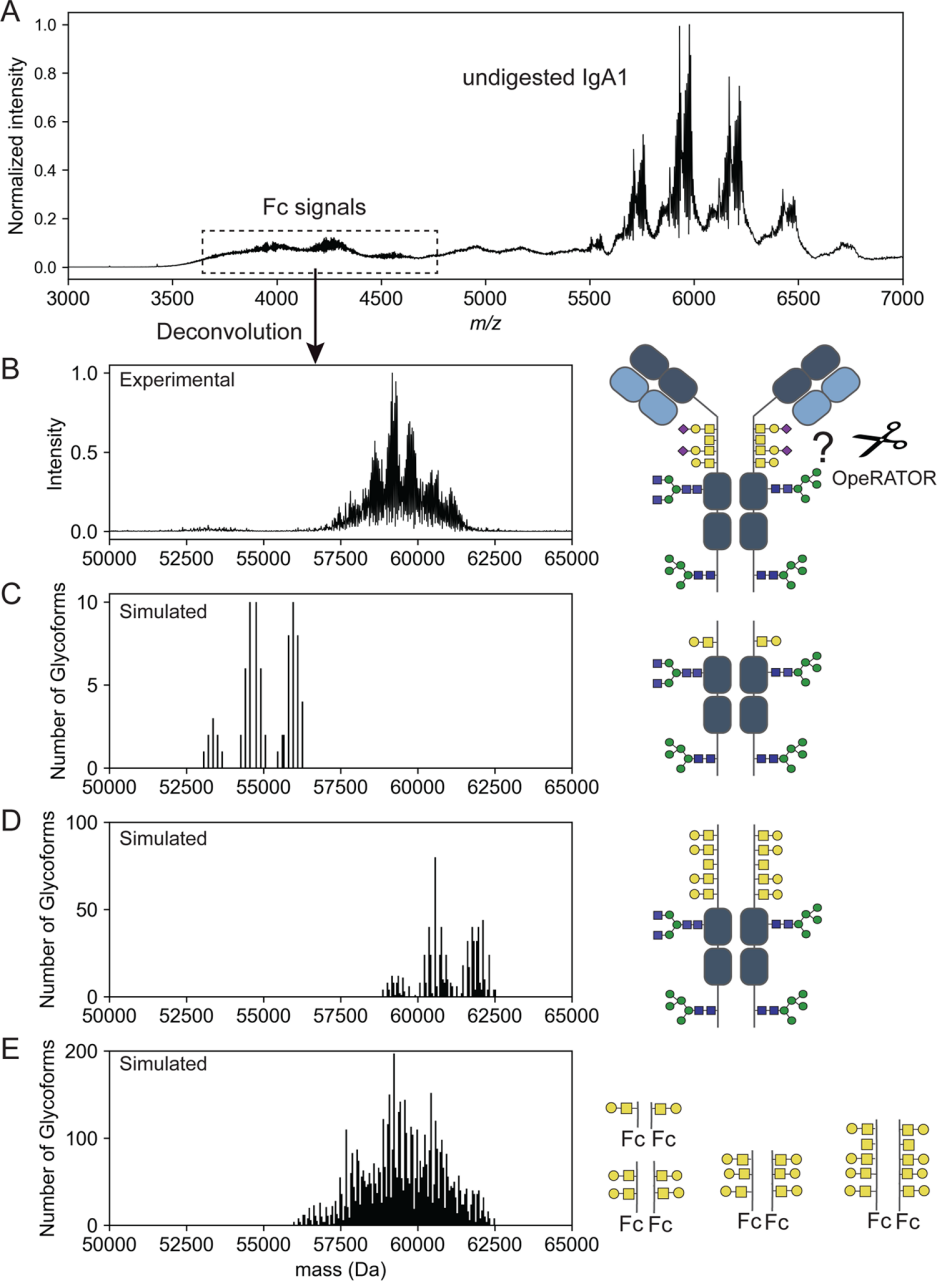
Experimental native MS spectra and simulated mass profiles of recombinant IgA1 partially digested by OpeRATOR. (A) Native mass spectrum of recombinant anti-CD20 IgA1 partially digested into Fc fragments (*m*/*z* 3500–4700) with residual undigested intact IgA1 (*m*/*z* 5500–7000). (B) Deconvoluted mass spectrum of the Fc ion signals from panel (A). (C) Simulated zero-charge mass spectrum of the Fc fragments taking into account only one missed cleavage (Ser243) bearing one core 1 *O*-glycan per each HR, (D) simulated zero-charge mass spectrum of the Fc fragments accounting for the full HR including Pro224 and with all *O*-glycosylation sites occupied, based on bottom-up glycoproteomics data presented in Figure 1, and (E) optimized simulated zero-charge mass spectrum matching best the experimental spectrum presented in panel (B), representing a population mixture of Fc fragments with variable HR lengths and variation in *O*-glycosylation occupancy.

As attempts to remove all *O*-glycan heterogeneity using *O*-glycosidase were not successful, we decided to take advantage of the specificity of the OpeRATOR enzyme, which cleaves the protein backbone *N*-terminally at core 1 *O*-glycans (HexNAc-Hex). It has been established that OpeRATOR cleaves IgA1 in the HR, producing Fab fragments with none or one missed cleavage, with the latter incorporating a core 1 *O*-glycan.^33^ Therefore, we rationalized that OpeRATOR might also digest IgA1 above the first residue containing a core 1 *O*-glycan in the HR (Ser243), generating an Fc fragment with just one *O*-glycan (Figure 3). We incubated the recombinant IgA1 with OpeRATOR and collected the Fc fragments, which were then mass analyzed by native MS (Figure 3A). Deconvoluted masses ranged surprisingly high, from 58 to 62 kDa (Figure 3B), while we estimated a theoretical mass of 51.714 kDa. This estimation included the Fc region with one missed cleavage (Ser243) harboring one core 1 *O*-glycan per HR and four *N*-glycans (taking the most abundant composition for each *N*-glycosite, i.e., N4H3 and N2H5 for N144 and N340, respectively). An additional mass in the experimental data of ∼2 to 5 kDa therefore remained unassigned, suggesting that a large population of the generated Fc fragments likely contained more missed cleavages.

#### Simulation of Mass Spectra Using Glycan Composition Data from Glycoproteomics Reveals the Coexistence of Fc Fragments with Varying HR Lengths Formed by OpeRATOR-Induced Cleavage

**Figure 4:**
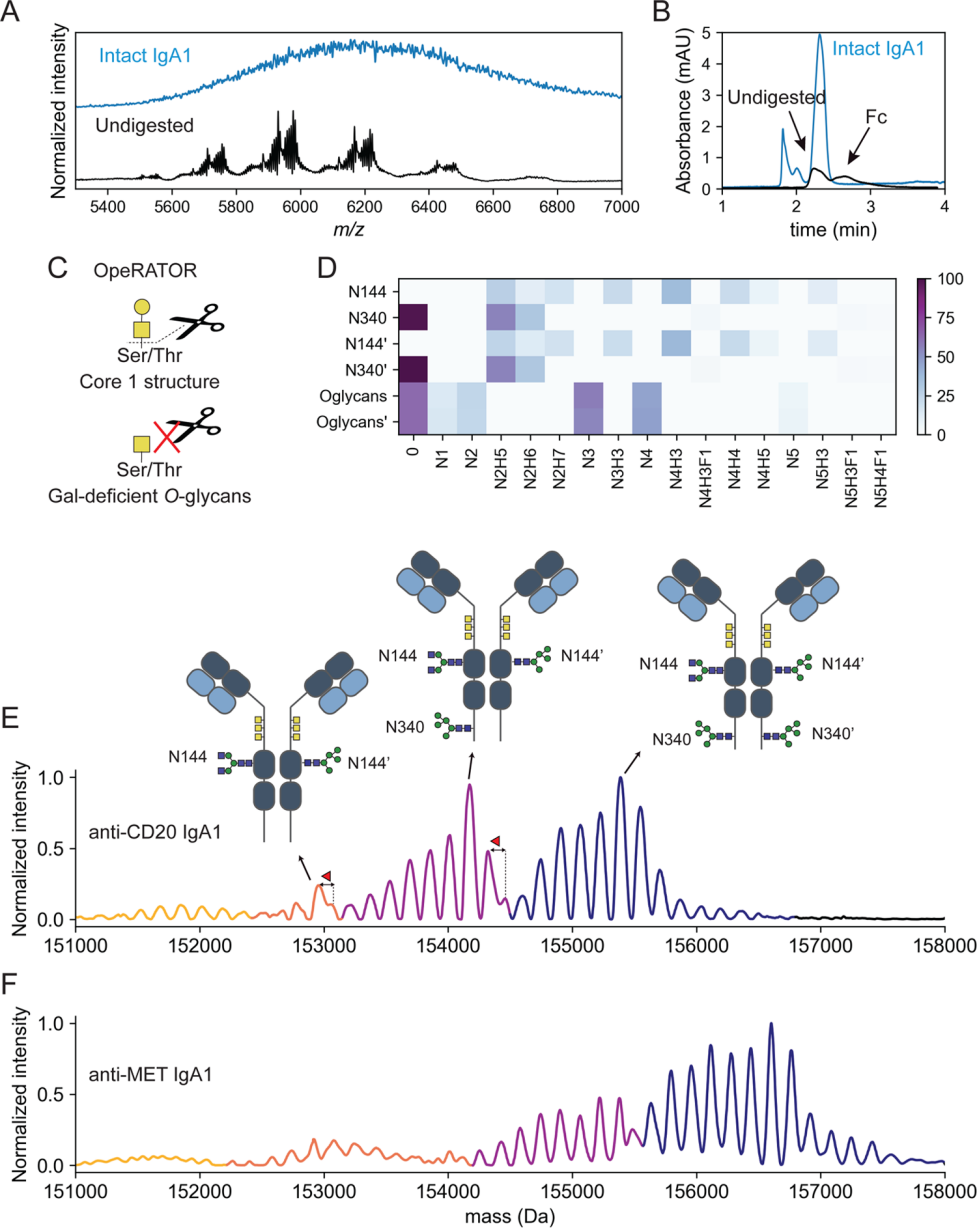
Resolving *N*-glycoforms of galactose-deficient (Gd) recombinant anti-CD20 and anti-MET IgA1. (A) Comparison of native mass spectrum of intact anti-CD20 IgA1 (from Figure 2A) vs undigested anti-CD20 IgA1 species remaining after OpeRATOR digestion (from Figure 3A). The MS data were acquired at 64 ms transient times (data acquired at other transient times are depicted in Figure S4). (B) SEC-UV of intact anti-CD20 IgA1 (blue) and after digestion of anti-CD20 IgA1 with OpeRATOR (black), used to fractionate the Fc fragments from the undigested IgA1. (C) Overview of the observed digestion specificity of OpeRATOR. (D) Heatmap of Gd anti-CD20 IgA1 based on *N*- and *O*-glycan compositions as determined by glycoproteomics. Deconvoluted native mass spectrum of (E) Gd anti-CD20 IgA1 and (F) Gd anti-MET IgA1. The mass distributions are colored based on the number of *N*-glycosites occupied with blue (4), purple (3), and orange (2). Yellow peaks represent IgA1 molecules with unoccupied HR. Peak-spacing differs by one Hex unit (162 Da) except when indicated, where the mass difference (146 Da) can be assigned to a fucose unit.

To verify the co-occurrence of multiple *O*-glycans in the HR of the Fc fragments (formed by OpeRATOR-induced cleavage), the Fc mass spectra were simulated based on all possible glycoform combinations extracted from the glycoproteomics data (Figure 3C–E). These data were compared with the experimental mass spectrum (Figure 3B). The first simulation was made with the assumption that OpeRATOR would cleave the Fc region at Ser243 (one missed cleavage), thus giving a backbone mass of 50,444 Da, to which the four *N*-glycans and two core 1 *O*-glycans were added. However, the simulated mass spectrum substantially underestimated the masses compared to the experimental Fc masses (Figure 3C). This indicated that OpeRATOR also cleaved above Ser243 in the HR, leaving more than one *O*-glycosylation site in the cleaved Fc region. Thus, in the next simulation, the assumption was made that OpeRATOR would cleave before the last *O*-glycosylation site (after Pro224), leading to a theoretical backbone mass of 52,738 Da, to which the masses of the four *N*-glycans and six core 1 *O*-glycans were added (Figure 3D). The simulated mass spectrum partially overlapped with the experimental spectrum (Figure 3B) but seemed to overestimate some masses. These simulations suggest that OpeRATOR might cleave the HR less specifically at multiple sites, leaving a variety of *O*-glycans on the Fc fragments. Therefore, the simulations were repeated using multiple Fc fragment masses with variable HR lengths, each containing one to several occupied *O*-glycosylation sites (Figure 3E). Through the latter simulation approach, an optimal agreement could be reached with the experimental data, thus implying that OpeRATOR does cleave recombinant anti-CD20 IgA1 at multiple sites, forming a mixture of Fc fragments with variable HR lengths, containing a varying number of *O*-glycans. This finding could be confirmed by purification of the OpeRATOR-cleaved Fc fragments using size exclusion chromatography (SEC), followed by bottom-up glycoproteomics (Figures 4B and S3).

#### OpeRATOR Does Not Cleave Galactose-Deficient *O*-GalNac IgA1 Glycoforms

Interestingly, when anti-CD20 IgA1 was incubated with OpeRATOR, we noticed that a substantial portion of IgA1 seemed to be resistant to cleavage (Figure 3A, labeled as undigested IgA1). Notably, the native mass spectrum of this “undigested” IgA1 was better resolved compared to that of untreated IgA1 (see Figures 2A and 3A). This observation aligns with the known specificity of OpeRATOR, which efficiently cleaves adjacent to core 1 *O*-glycans but is less effective when Tn antigen *O*-glycans (i.e., galactose-deficient (Gd) *O*-glycans) are present.^32,34,35^ Indeed, our bottom-up glycoproteomics data (Figure 1) revealed that approximately 8–10% of the *O*-glycans in recombinant anti-CD20 IgA1 are galactose-deficient, making this population likely resistant to OpeRATOR cleavage. In agreement, using SEC, we estimated that about 12% of the IgA1 remained nondigested, representing Gd *O*-GalNac IgA1 glycoforms (Figure 4B,C). These results indicate that the heterogeneity observed in recombinant anti-CD20 IgA1 at the intact level mainly originates from the huge heterogeneity of the *O*-glycans in the HR, as we were able to obtain well-resolved native mass spectrum in their absence.

#### Resolved Mass Spectrum of Intact Galactose-Deficient *O*-GalNac IgA1 Glycoforms Allows Glycoforms Assignment

To assign the masses in the well-resolved mass spectra of OpeRATOR-resistant anti-CD20 IgA1 to glycan structures, we constructed a site-specific library based on Gd *O*-glycoforms and *N*-glycan compositions derived from the glycoproteomics data and matched these with the experimental masses. We employed a probabilistic approach, where site-specific fractional abundances from the glycoproteomics data were normalized by the peak height of intact glycoforms, generating a glycan heatmap (Figure 4D).^13^ In the best performing models, the HR was either unoccupied or carried 3–4 HexNAc *O*-glycans. We assumed that the N340 site was either unoccupied or contained N2H5 and N2H6 *N*-glycans. N144 was fully occupied and harbored a larger number of different glycoforms with N4H3 being the most abundant. According to this model, the IgA1 molecules should be composed of at least three distinct populations, all carrying ∼6 HexNAc in the HR, but with varying *N*-glycosylation: a fraction (∼10%) with both N340 sites unoccupied, a fraction (∼45%) with only one N340 site occupied, and a fraction (∼45%) with both N340 sites occupied (Figure 4E). As this model provides excellent agreement with the experimental data, we unambiguously conclude that intact recombinant IgA1 is asymmetrically glycosylated in the tailpiece.

Given that this asymmetric glycosylation in IgA1 has never been shown before, we aimed to validate our findings by analyzing a second recombinant IgA1, termed IgA1–5d5, which targets the mesenchymal–epithelial transition factor (MET), also produced in HEK293 cells. The anti-MET IgA1 was incubated with OpeRATOR and also showed a digestion-resistant IgA1 population. The deconvoluted native mass spectrum of this construct, shown in Figure 4F, closely resembled the mass spectrum shown in Figure 4E, albeit with some differences in intensities and masses. A larger fraction of anti-MET IgA1 had all four *N*-glycosylation sites occupied compared with anti-CD20 IgA1. In more detail, we estimated the populations to be 10/30/60% in anti-MET IgA1 and 10/45/45% in anti-CD20 IgA1. With these results in hand, we next aimed to explore whether these unexpected asymmetric glycosylation features were unique for the Gd *O*-GalNac IgA1 glycoforms or also present in the entire IgA1 pool.

### Electron-Capture Charge Reduction (ECCR) Assists in Assigning Glycan Compositions on Glycoproteins

Due to the high congestion of ion peaks from the cooccurring glycoproteoforms within a narrow *m*/*z* window, the native mass spectrum of intact IgA1 appeared unresolved (Figure 2A). Without using enzymes to remove the glycans effectively, an alternative approach to resolving this congestion is to reduce the charges on these ions to increase the *m*/*z* spacing between consecutive charges. In native MS, charge reduction can be performed by adding additives, such as triethylamine (TEA)^36^ or imidazole^37^ in the spray solution (i.e., proton sponges). Charges on the ions can also be reduced in the gas phase, for instance, by using proton-transfer charge reduction (PTCR). PTCR is a tool for reducing spectral congestion and has been primarily used in top-down proteomics^38^ and in combination with native MS.^39^ Additionally, electron transfer and electron capture are viable methods for manipulating the charge states of the native protein complexes. Recently, electron-capture charge reduction (coined ECCR) was used in native MS to resolve highly heterogeneous systems such as adeno-associated virus (AAVs), viral FLIP, and SARS-CoV-2 spike protein timer construct.^14,40^ Limited charge reduction using electron transfer dissociation (ETD) has also been shown to facilitate the analysis of other highly heterogeneous protein complexes.^41−43^ By optimizing ECCR conditions, a highly protonated protein or protein complex could capture free electrons without apparent dissociation, lowering the charge states and shifting the ions to higher *m*/*z*, where the spacing between signals within charge states is larger.^44−46^ However, as the resolving power of an Orbitrap mass analyzer decreases as a function of the , the peaks become broader at higher *m*/*z*, reducing mass accuracy.^47^

**Figure 5:**
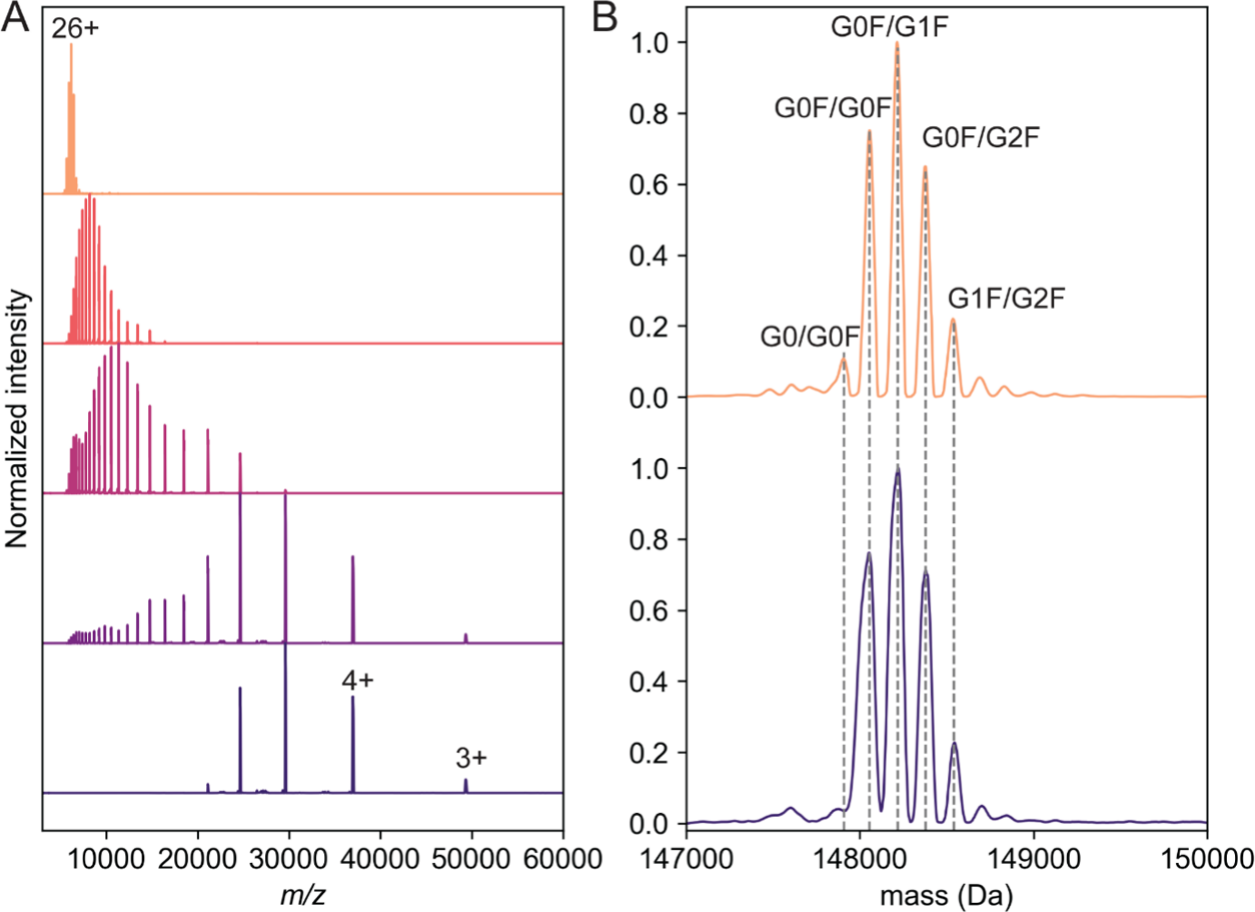
Electron-capture charge reduction (ECCR) native mass spectrum of IgG1. (A) ECCR native mass spectrum of trastuzumab at increasing charge-reduction conditions using 128 ms transient time (in Table S2, the used ECCR parameters are provided). Numbers annotate the charge. (B) Deconvoluted mass spectrum of nonreduced intact (from top spectrum in panel (A)) and charge-reduced intact trastuzumab (from bottom spectrum in panel (A)) with mass-resolved *N*-glycosylations, revealing that ECCR does not affect the glycoform profiles of the intact IgG1.

We first investigated whether applying ECCR would affect the “labile” glycans attached to antibodies, focusing initially on the recombinant anti-HER2 IgG1 trastuzumab. The native mass spectrum of trastuzumab displayed the expected charge-state distribution, ranging from 27+ to 21+ (Figure 5A top, B).^48^ We then tuned the ECD cell to enable ion–electron reactions. By setting the ion optics to reduce the kinetic energy and the HCD cell to maximize ion transmission, we were able to charge-reduce trastuzumab down to the 3+ charge state (Figure 5A), at *m*/*z* ∼ 50,000. Deconvolution of the native mass spectrum qualitatively and quantitatively showed the expected glycosylation pattern for trastuzumab (Figure 5B). We found that not only the glycoform compositions but also the relative glycoform intensities remained identical upon charge reduction (Figure 5B), demonstrating that ECCR does not induce substantial glycan losses and can thus be used to mass-analyze glycosylated proteins.

### Exposing *N*-Glycan Asymmetry in Recombinant IgA1

**Figure 6:**
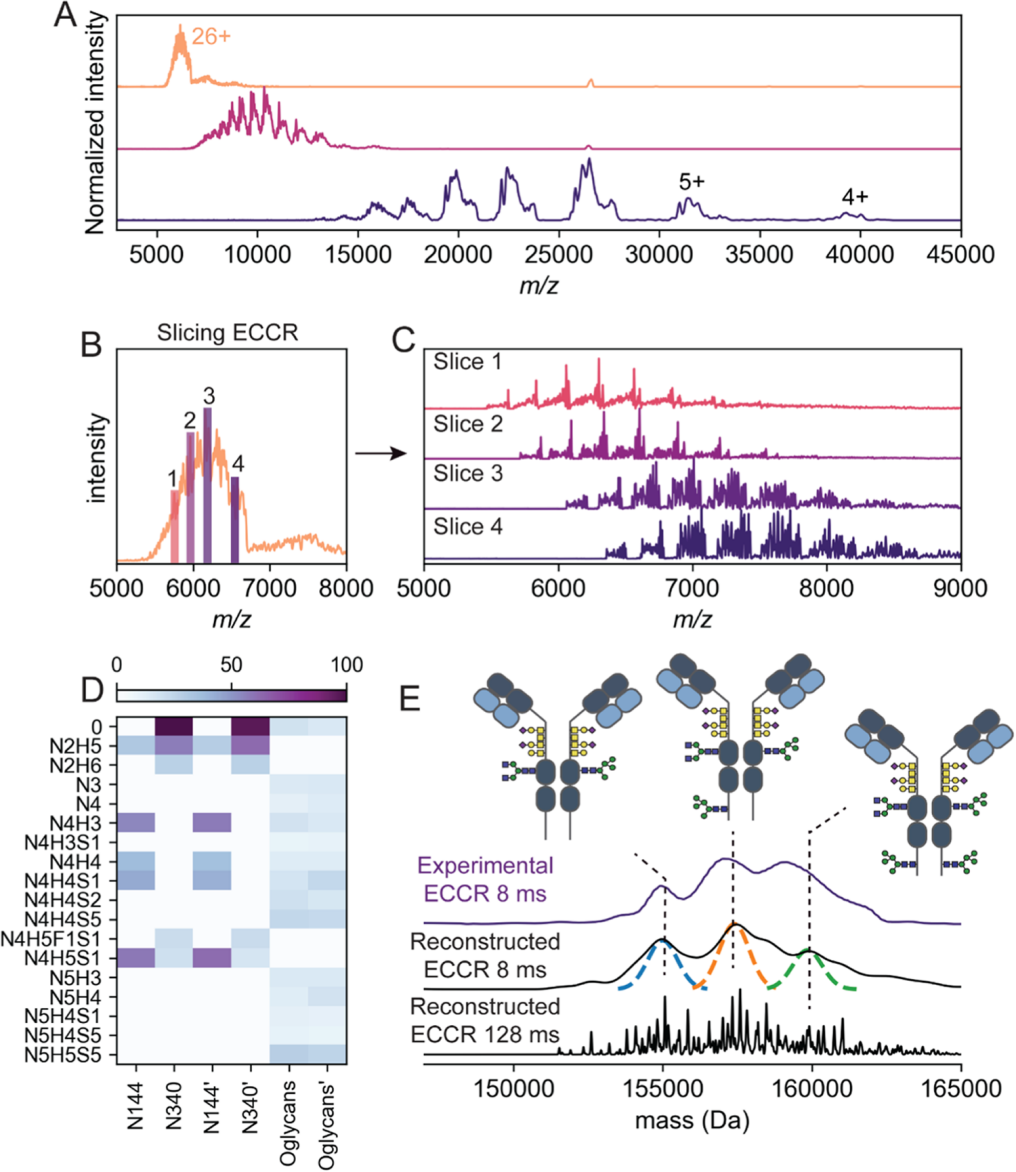
Electron-capture charge-reduction (ECCR) native mass spectrum of anti-CD20 IgA1. (A) ECCR for intact IgA1 acquired using a fixed transient time of 8 ms (in Table S3, the used ECCR parameters are provided). Numbers indicate the charges. (B) Slicing ECCR by quadrupole-selecting small *m*/*z* windows in the native mass spectrum provides increased resolving power and reveals the asymmetry in *N*-glycan occupancy. (C) ECCR of selected *m*/*z* windows from panel (B) acquired at a 32 ms transient. (D) Heatmap of the whole population of IgA1 glycoforms based on *N*- and *O*-glycan compositions as determined by glycoproteomics. (E) Comparison of the experimental deconvoluted ECCR spectrum versus the reconstructed ECCR. For the reconstructed ECCR profiles, masses derived from the slicing ECCR as well as their relative abundances were used as input to simulate a native mass spectrum at two different transient times (8 and 128 ms). The blue, orange, and green Gaussians represent the glycoform populations with two, three, and four *N*-glycans.

Next, we explored ECCR to determine whether the observed *N*-glycosylation asymmetry was also present in the full population of intact recombinant IgA1 molecules (Figure 6A). A similar 80% charge reduction as achieved for trastuzumab could be obtained, bringing the initial average charge of 26+ down to 7+. As hoped, the charge-reduced mass spectrum exposed some resolved charge states, with each one being dominated by around 3–4 peaks. Next, a slicing ECCR approach was used (Figure 6B), isolating multiple smaller *m*/*z* windows as precursors for ECCR. This approach resulted in much less dense mass spectrum, allowing us to record the charge-reduced mass spectrum with higher resolution (128 ms) and reveal distinct glycoforms, including those differentiated by a single monosaccharide unit (Figure 6C).

To assign these masses to glycoforms, we first constructed a site-specific library using the top 15 *O*-glycoforms and *N*-glycoforms derived from the glycoproteomics data (Figure 6D). Then, based on the masses and relative abundances of all deconvoluted masses from the slicing ECCR, we reconstructed a native mass spectrum that most closely resembled the experimental ECCR spectrum, revealing three distinct mass distributions (Figure 6E). However, the resolution was insufficient to assign specific glycoforms to the residual masses. By relying on the resolved masses obtained from the slicing ECCR approach, we simulated a mass spectrum at a resolution (128 ms) capable of distinguishing closely related glycoforms (Figure 6E). We rationalized that the most abundant masses in each of the three glycoform mass distributions corresponded best to IgA1 molecules with either two, three, or four *N*-glycosylation sites occupied. Like what we observed for the Gd *O*-GalNac IgA1 glycoforms fraction, the whole population of recombinant anti-CD20 IgA1 contained molecules with two, three, or four occupied *N*-glycosites, confirming the asymmetric *N*-glycosylation of the tailpiece.

## Conclusions

Nearly all therapeutic antibodies clinically used originate from the IgG class, while research into and clinical use of IgA has been much more limited.^23−25^ In contrast to the single *N*-glycosylation site per heavy chain in IgG, IgA has 2 or 4–5 *N*-glycosylation sites in the heavy chain of IgA1 and IgA2, respectively. Consequently, IgA has a more heterogeneous *N*-glycosylation pattern, which supposedly contributes to the shorter half-life of IgA.^49^ Here, we developed a mass spectrometry workflow to dissect the glycan structures that assemble together in recombinant IgA1. Using bottom-up glycoproteomics, we annotated more than 150 *O*-glycan compositions. According to this data, site N144 was fully occupied, primarily by diantennary complex-type glycans (53%), while site N340 showed 70% occupancy, predominantly with high-mannose glycans. Performing standard native MS on intact IgA1 resulted in broad, unresolved mass profiles. While using enzymes to induce the selective cleavage of *N*- and/or *O*-glycans is sometimes sufficient to enable glycoprofiling by native MS,^10,12,50−52^ the mass spectrum of IgA1 remained largely unresolved even with combinations of these glycosidases. Next, we used OpeRATOR, an enzyme that cleaves *N*-terminally core 1 glycosites,^15,33^ to cleave IgA1 into smaller Fc and Fab fragments, aiming to generate Fc fragments free of *O*-glycan heterogeneity. However, OpeRATOR instead introduced additional heterogeneity by generating a mixture of Fc fragments with varying HR lengths, each harboring multiple *O*-glycans. Moreover, a sizable fraction (∼12%) of IgA1, was resistant to cleavage. This substantial fraction was found to originate from IgA1 molecules harboring galactose-deficient *O*-glycoforms. Indeed, OpeRATOR preferentially cleaved asialylated core 1 *O*-glycans, with almost no activity toward galactose-deficient *O*-glycans.^32,34,35^ The native mass spectrum of enzymatically resistant IgA1 molecules displayed well-resolved peaks. Using a simulation approach, based on the *O*- and *N*-glycan compositions derived from bottom-up glycoproteomics data, we were able to assign glycan compositions to the experimental masses observed in these mass spectra. Our analysis revealed that galactose-deficient IgA1 molecules consisted of three distinct glycoform populations: a very small fraction with both N340 sites unoccupied and two roughly equally populated glycoform distributions with one or two N340 sites occupied. To reveal if these features were also present in the entire IgA1 pool, we used ECCR native MS. We found that the whole population of IgA1 was composed of molecules with two, three, and four *N*-glycosites occupied. Comparing anti-CD20 IgA1 to anti-MET IgA1, the latter showed a larger population with all four *N*-glycosylation sites occupied. Our findings provide evidence that the IgA1 assembly is not stochastic and that the variable region modulates the C-terminal *N*-glycosylation occupancy.

The asymmetric occupancy of *N*-glycosylation sites could potentially be functionally relevant, as alterations in glycosylation patterns, particularly during production, can enhance immunogenicity and clearance.^20^ Moreover, the IgA1 tailpiece, harboring the N340 site, is involved in binding to the J-chain and the PIGR receptor, and may thus also affect IgA1 polymerization.^53,54^

This study focuses on recombinant IgA1 produced in human HEK293 cells, as this provides a tangible route to produce therapeutic IgA1-based antibodies. Still, it may be relevant to briefly discuss the main differences observed here between this recombinant IgA1 and, for instance, IgA1 present in human serum, especially in relation to its glycosylation features. Most notably, in serum IgA1 from healthy donors, the tailpiece N340 site in typically fully occupied (preventing any potential asymmetry), mostly with diantennary and extended, fully sialylated glycans.^17,55,56^ Moreover, the *O*-glycan sites in serum IgA1 are also very heterogeneously glycosylated, and not always fully occupied.^20,57,58^ These differences highlight the importance of being able to fully characterize recombinant IgA1, as well as assisting recombinant production to make more “serum-alike” IgA1 therapeutics. The multimodal MS approaches presented here should therefore be beneficial in supporting the future development of therapeutic IgAs as alternatives to recombinant IgGs.

## Experimental Section

### Enzymatic Digestion and Sample Preparation for Bottom-Up Proteomics

IgA (5 μg) was dissolved in 20 mM TRIS (pH 8.5) and reduced with 5 mM TCEP at 60 °C for 10 min, and cysteines were alkylated with 20 mM chloroacetamide. Sodium dodecyl sulfate (1% w/v) was added, followed by trypsin digestion (1:50 w) overnight at 37 °C. The reaction was stopped with 1% TFA, and peptides were desalted using an OASIS HLB 96-well μElution plate (Waters) and then dried in vacuo.

### Liquid Chromatography-Mass Spectrometry (LC–MS)/MS-Based Glycoproteomics

Peptides were separated on an Ultimate-3000 HPLC system (Thermo Fisher Scientific) coupled to an Orbitrap Eclipse Mass Spectrometer (Thermo Fisher Scientific) operated in positive mode. Peptides were trapped on Acclaim Pepmap 100 C18 (5 mm × 0.3 mm, 5 μm, Thermo Fisher Scientific) and separated on an in-house packed C18 analytical column (Reprosil 2.4 μm, 75 μm × 50 cm) at 40 °C. Mobile phase A consisted of 0.1% FA and mobile phase B consisted of 0.1% FA in 80% ACN. The LC gradient was supplied at 0.300 μL·min^–1^ over a 43 min gradient (4–55% B). MS analysis was performed using the stepped-HCD and EThcD methods. MS1 scans were acquired at 120,000 resolution with a 350–2000 *m*/*z* range using a maximum injection time (IT) of 246 ms. The AGC target, 400,000; normalized AGC target, 100%. MS2 spectra were acquired with a resolution of 30,000, a 120–4000 *m*/*z* scan range, 50 ms maximum IT, 50,000 AGC target, 100% normalized AGC target, and 29% HCD energy. An additional MS2 scan, using either HCD stepping or EThcD, was triggered if the initial scan contained at least three glycan oxonium ion signals (Table S1). Stepped-HCD settings included 60,000 resolution, 120–4000 *m*/*z* scan range, 150 ms maximum IT, 50,000 AGC target, 100% normalized AGC target, and HCD energies of 20 and 40%. Similar settings were used for EThcD, but with a 100,000 AGC target and 200% normalized AGC. Calibrated charge-dependent ETD parameters were used, and supplemental HCD energy of 15% was applied.

### Glycoproteomics Data Processing

Glycopeptides were identified from raw data using PMI-Byonic. For the *N*-glycan search: the protein database consisted of a known sequence of the monoclonal IgA1, and the *N*-glycan database consisted of 279 common human *N*-glycans. For the *O*-glycan search: the protein database consisted of a single amino acid stretch comprising the hinge region of IgA1 (HYTNPSQDVTVPCPVPSTPPTPSPSTPPTPSPSCCHPRLSLHR) and the *O*-glycan database consisted of five compositions (N1, N1H1, N1S1, N1H1S1, and N1H1S2) and nine *O*-glycans were allowed per peptide. For all searches, fully specific digestion was used, three missed cleavages were allowed, and precursor and fragment mass tolerances were set to 20 ppm. Quantification of the glycopeptides was performed using PMI-Byologics. PSMs were manually curated and filtered based on their score (>150) and exported. Further processing was performed using R.

### Enzymatic *N*- and *O*-Glycan Removal

*N*-glycans were released from the intact IgA1 using PNGase F (Roche Applied Science, Mannheim, Germany) by incubation of one unit of PNGase F with 5 μg of IgA1 overnight at 37 °C with shaking at 750 rpm. To remove *O*-glycans, 5 μg of IgA1 were treated overnight with five units of SialEXO (Genovis, Llund, Sweden) and one unit of *O*-Glycosidase (New England Biolabs, Ipswich, MA) at 37 °C with shaking at 750 rpm. The simultaneous removal of *N*- and *O*-glycans was achieved by incubation of 5 μg of IgA1 with five units of SialEXO, one unit of *O*-Glycosidase, and one unit of PNGase F at 37 °C overnight and with shaking at 750 rpm.

### Native MS

Native MS was performed on a Q-Exactive UHMR Orbitrap mass spectrometer (Thermo Fisher Scientific, Bremen, Germany) equipped with a nanoelectrospray source. IgA1 samples (1–3 μM in 200 mM ammonium acetate, pH 7.5) were desalted using μZeba spin 7K columns (Thermo Fisher Scientific). A 1–4 μL aliquot was loaded into gold-coated borosilicate capillaries, and ions were generated with a 1.2–1.5 kV potential. The source capillary temperature was 250 °C, S-lens RF was set to 200, and the ion transfer target and detector optimization were set to “high *m/z*”. In-source trapping was set to a desolvation voltage of −100 V, and ions were transported to the HCD cell with an injection energy of 100 V for desolvation. The ion transfer optics (injection flatapole, interflatapole lens, and bent flatapole) were set to 8, 6, and 8 V, respectively. Nitrogen was used as a collision gas and the ultrahigh vacuum (UHV) readout was in the range of 2 × 10^–9^ to 4 × 10^–9^ mbar. Data was acquired at various resolution settings corresponding to 8, 16, and 32 ms transients. Mass deconvolution was performed using UniDec v.6.0.4.^59^

### Online Native Size Exclusion Chromatography

Native SEC analysis was performed using an Agilent 1290 Infinity HPLC system equipped with an ACQUITY UPLC Protein BEH SEC 200 Å Column (4.6 × 300 mm, 1.7 μm particle size; Waters), using 100 mM ammonium acetate, pH 6.8, as the mobile phase. After column elution, the flow was split in a 1:65 ratio between the Q-Exactive UHMR Orbitrap mass spectrometer (Thermo Fisher Scientific, Bremen, Germany) and an Agilent 1200 Infinity variable wavelength detector set at 280 nm.

### Electron-Capture Charge Reduction

A Q-Exactive UHMR Orbitrap mass spectrometer (Thermo Fisher Scientific, Bremen, Germany) equipped with an ExD TQ-160 (e-MSion) cell was used for the electron-capture charge-reduction (ECCR) experiments. The ion transfer optics (injection flatapole, interflatapole lens, bent flatapole) were set to 4, 2, and 2 V, respectively. We modified the ion transfer optics to decrease the kinetic energy of electrosprayed ions to increase the reaction time in the ExD cell.^40^ To charge-reduced trastuzumab, in-source trapping was set to a desolvation voltage of −150 V, HCD cell to 120 V, and fixed injection time to 250 ms. Data was collected for 1 min and the spectra averaged. The transient time was set at 128 ms. The trapping gas pressure was set at 4, and the UHV readout was around 2 × 10^–10^ mbar. The filament current was set to 2.3 A and the ExD cell voltages corresponding to L2, LM3, L4, FB, LM5, and L6 were tuned to obtain spectra at different charge-reduction degrees (Table S2). Other UHMR parameters related with the timing and voltages in the HCD cell were also optimized to transmit high *m*/*z* ions to the C-trap (Table S2). Specifically, we increased the HCD purge time to the C-trap, as ions with higher *m*/*z* require more time to exit the HCD cell and reach the C-trap, following a time-of-flight process. The voltage that is applied to the C-trap exit lens when ions exit the HCD cell to the C-trap was also tuned together with the HCD multipole DC and field gradient. ECCR for IgA1 was acquired using 8 ms transient time, and the ExD cell voltages and UHMR parameters are listed in Table S3. For slicing ECCR, we divided the 5500–7600 *m*/*z* region of the native mass spectra of IgA1 into twenty-one 250 *m*/*z* windows. The ion transfer optics (injection flatapole, interflatapole lens, bent flatapole) were set to 8, 6, and 8 V, respectively. In-source trapping was set to −150 V, HCD cell to 80 V, injection time 250 ms, and AGC fix to 3e6. The filament current was set to 2.3 A and the ExD cell voltages corresponding to L2, LM3, L4, FB, LM5, and L6 were set to −44, 8, 9, 1, 8, and −44 V, respectively. The trapping gas pressure was set at 3, and the UHV readout was around 1.7 × 10^–10^ mbar. Data were acquired for 1 min at 32 ms transients, and spectra were averaged over the time.

### Slicing ECCR Data Reconstruction of Pseudonative MS Spectrum

Charge-reduced slices were first deconvolved. The peak ion intensities were multiplied by the injection time to estimate the number of ions. The list of masses and intensities were used as an input for simulation of a pseudonative mass spectrum using published python scripts.^60^

### Integration of Bottom-Up Glycoproteomics and Native MS and Slicing ECCR

To assign glycan structures to masses derived from native MS or slicing ECCR, we constructed a probabilistic model using bottom-up glycoproteomics data. Initially, we generated all possible glycan combinations using the Cartesian product of the bottom-up-determined glycan composition of the four glycosites (N144, N340, and *O*-glycosite). We then consider a 6-site independent model (N144, N144′, N340, N340′, O-glycosite, O-glycosite’). Let *S* = {*S*_1_, *S*_2_, ···, *S_n_*} be the set of glycosites, and let **I** = (*i*_1_, *i*_2_, ···, *i_n_*) denote the combination of indices of glycans at each site, the mass of a glycoform *M*(**I**) can be calculated as the sum of the masses of selected glycans
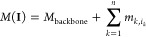
where *M*_backbone_ is the protein backbone mass corrected for disulfide bridges, *n* is the number of glycosites, and *m_k_*,_*ik*_ is the mass of the *i*_*k*_th modification at site *S*_*k*_. The intensity of a particular glycoform was calculated as the product of the individual probabilities of each site for that combination
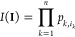
where *p_k_*,_*ik*_ is the normalized relative abundance of the *i*_*k*_th glycan at site *S*_*k*_. It should be noted that using all possible *N*-glycan and *O*-glycan structures derived from BU glycoproteomics, we calculated approximately 2 × 10^11^ possible glycoforms. Then, we performed a mass-to-mass matching using a mass filter (5 Da) and product intensity filter, selecting the top-5 best matching solutions based on combined probability. To better reflect the relative intensities of the native mass spectrum, the intensity of each particular glycoform *I*(**I**) was normalized by the peak height of the native mass spectrum of the assigned glycoform and plotted as heatmap.

### Generation of Fc Fragments by Hinge-Region Digestion of IgA1s

The *O*-glycopeptidase OgpA from Akkermansia municiphila (OpeRATOR, Genovis, Llund, Sweden) was used to digest 50 μg of IgA1 and generate Fab and Fc subunits. Digestion was performed by adding 1 unit of SialEXO (Genovis) and 1 unit of OpeRATOR per μg of IgA1. SialEXO is a sialidase cocktail, which removes sialic acids from the *O*-glycans, and boosts the efficiency of hinge digestion by OpeRATOR that is more active toward nonsialylated core 1 *O*-glycans. Samples were incubated overnight at 37 °C while being shaken at 750 rpm.

### Collection of Fc Fragments Following IgA1 Hinge-Region Digestion

The digested IgA1 was captured using a CaptureSelect IgA affinity matrix (Thermo Fisher Scientific). 35 μL bead slurry was added directly to Pierce spin columns with screw cap (Thermo Fisher Scientific). The beads were then repeatedly washed with 150 μL of PBS by centrifugation at 500*g*. After the third wash, a plug was inserted to the bottom of the individual spin columns, and the digested IgA1 (∼150 μL) was added to the beads. Samples were then incubated for 1 h while being shaken at 750 rpm at room temperature (RT). Following the incubation, the plugs were removed from the spin columns, and the flowthrough containing Fab fragments that are not retained by the beads was collected by centrifugation for 1 min at 500*g*, RT. The beads were then washed three times with 200 μL of PBS and two times with 200 μL of milli-Q water, by centrifugation for 1 min at 500*g*. Lastly, 100 μL of Glycine-HCl (0.1M, pH 2.7) was added to elute Fc fragments from the beads. After incubation for 10 min with shaking at 750 rpm (RT), the Fc subunits were collected by centrifugation for 1 min at 1000*g*.

## Supporting Information Available

Oxonium ion signals used to trigger MS2 scans in glycoproteomics experiments; parameters used for ECCR experiments; bar plots depicting the normalized abundance of *N*-glycans at the N340 and N144 sites in anti-CD20 IgA1; comparison between the masses and relative intensities of *O*-glycoforms annotated by Byonic with deconvoluted mass spectra; orbitrap-based native mass spectrometry of anti-CD20 IgG1 and IgA1 at different transient recording times; *O*-glycoproteomics analysis of anti-CD20 IgA1 after digestion with OpeRATOR and trypsin; and native mass spectrometry of recombinant anti-CD20 IgA1 after incubation with OpeRATOR (PDF)
