# Supplementary material for: Asymmetric N-Glycosylation in the Tailpiece of Recombinant IgA1
Source: J Am Chem Soc. 2024 Dec 6;146(50):34720–32. doi: 10.1021/jacs.4c13156 (PMC11664498; doi:10.1021/jacs.4c13156)
Supplement: Supplementary file 1 — ja4c13156_si_001.pdf [file ja4c13156_si_001.pdf]

Supporting Information

Asymmetric *N*-glycosylation in the tailpiece of recombinant IgA1

Manuel David Peris-Díaz [a,b], Evolène Desligniѐre[a], Shelley Jager[a], Nadia Mokiem[a], Arjan Barendregt[a], Albert Bondt[a], Albert J. R. Heck [a]

[a] Biomolecular Mass Spectrometry and Proteomics, Bijvoet Center for Biomolecular Research and Utrecht Institute for Pharmaceutical Sciences, Utrecht University, Utrecht 3584 CH, The Netherlands

[b] Department of Chemical Biology, Faculty of Biotechnology, University of Wrocław, F. Joliot-Curie 14a, 50-383 Wrocław, Poland

Table S1. Oxonium ion signals considered to trigger additional MS2 scan in the bottom-up glycoproteomics experiments. Glycan compositions are abbreviated to single letter codes, where H is hexose, N is N-acetyl hexosamine, P is phosphomannose, F is fucose, S is sialic acid.

Compound name	***m/z***
H	127.039
H	145.0495
H	163.0601
P	243.0264
P2	405.0793
N	138.055
N	168.0655
N	186.0761
N	204.0847
S	274.0921
S	292.1027
N1H1	366.1395
N2	407.166
N1H1F1	512.1974
N1H1S1	657.2349

Table S2. ExD cell voltage parameters used for the ECCR experiment of trastuzumab in Figure 5A.

**L1(V)**	**L2(V)**	**LM3(V)**	**L4(V)**	**FB(V)**	**LM5(V)**	**L6(V)**	**L7(V)**	**HCD purge time (ms)**	**C-trap exit lens purge (V)**	**HCD multipole DC purge (V)**	**HCD field gradient purge (V)**
0.2	2.8	-11	2.2	-2.3	-9.4	1.5	-0.4	5	2	3	40
0	-25	6	7	1	6	-25	0	15	2	3	40
0	-44	11	12	1	11	-44	0	15	-2	3	60
0	-44	15	20	1	15	-44	0	15	-3	4	60
0	-60	19	22	2.5	17	-60	0	15	-4.5	4	60

Table S3. ExD cell voltage parameters used for the ECCR experiment of IgA1 in Figure 6A.

L1(V)	L2(V)	LM3(V)	L4(V)	FB(V)	LM5(V)	L6(V)	L7(V)	HCD purge time (ms)	C-trap exit lens purge (V)	HCD multipole DC purge (V)	HCD field gradient purge (V)
0.2	2.8	-11	2.2	-2.3	-9.4	1.5	-0.4	5	2	3	40
0	-44	8	9	1	8	-44	0	5	2	3	40
0	-60	21	21	1.1	17	-60	0	30	-2.5	6	100

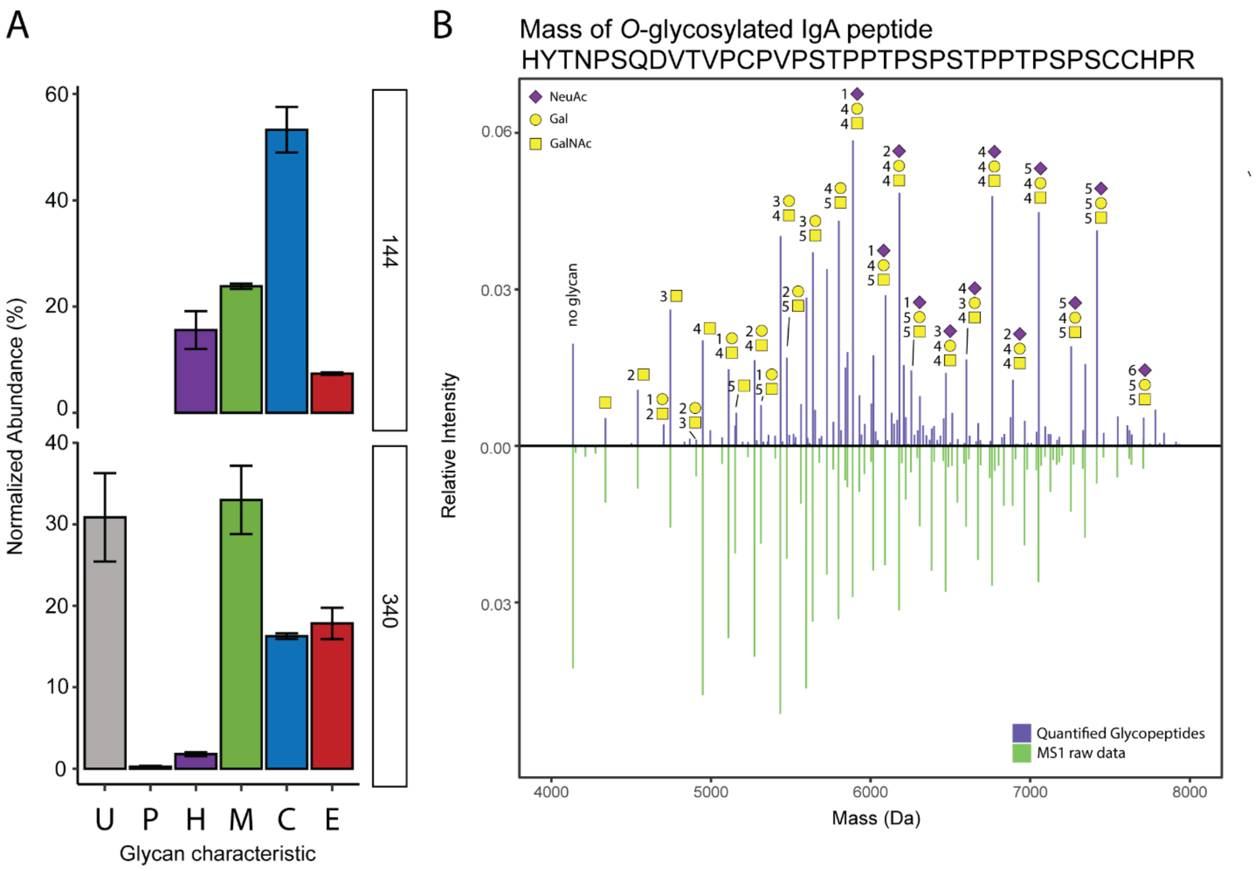

Figure S1. A) *N*-glycan characteristics for each of the two *N*-glycosylation sites of recombinant anti-CD20 IgA1 produced in HEK293 cells, derived from bottom-up glycoproteomics data, where U is unoccupied, P is phosphomannose, H is hybrid, M is high-mannose, C is complex and E is extended glycans (complex glycans with 5 or more HexNAc residues). At the top the data for N144 are depicted, at the bottom those for N340. The bars represent the sum of the individual glycopeptide intensities, averaged over two injection replicates. Error bars represent the standard deviation between the two injection replicates. **B)** Comparing the masses and relative intensities of the different glycoforms of the *O*-glycosylated tryptic peptide covering the hinge region of the anti-CD20 IgA1. The sequence of the tryptic peptide is given at the top, which has a mass of 4135.88 Da, when no *O*-glycans are attached. In the top panel, the calculated mass and relative intensities of the PSMs annotated by Byonic, while the bottom panel shows the Xtract deconvoluted MS1 spectra in the region where the glycoforms of this tryptic peptide elute (retention time window: 27.59-34.72 minutes). The most abundant glycoforms have been annotated with their composition. This figure clearly shows that all dominant glycoforms have been identified and quantified in the bottom-up search, as it matches the MS1 data.

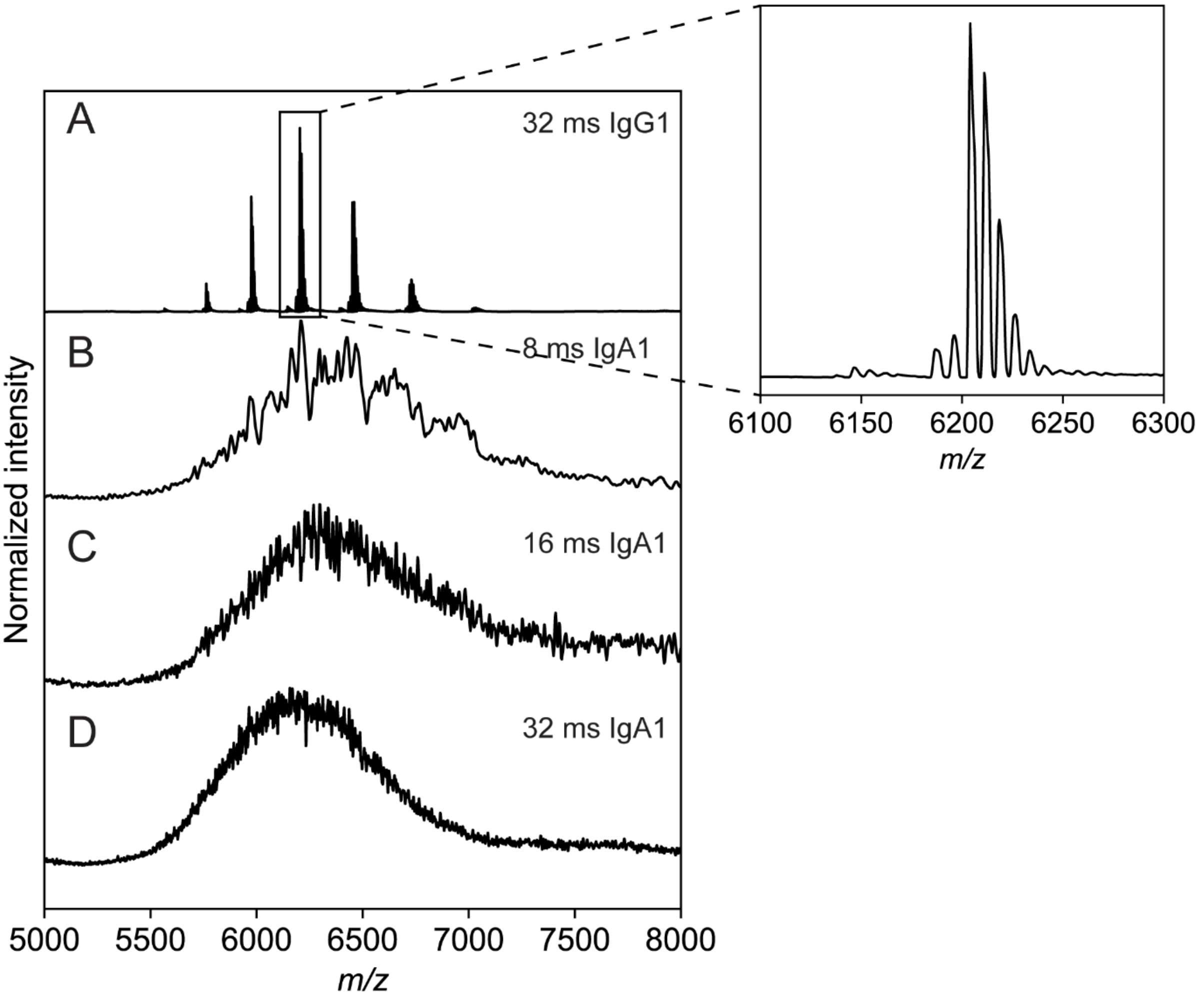

Figure S2. Orbitrap-based native mass spectrometry of anti-CD20 IgG1 and IgA1 at different transient recording times. A) IgG1 acquired at 32 ms displays well-resolved charge states and even resolves the glycoproteoform profile. Zoomed-in view of the 26+ ions from IgG1 acquired at 32 ms shows resolved glycoforms. In contrast, the native mass spectra of IgA1 are not resolvable at B) 8ms, C) 16 ms, and D) 32 ms transient times, due to the heterogeneity of the diverse palette of overlapping proteoforms.

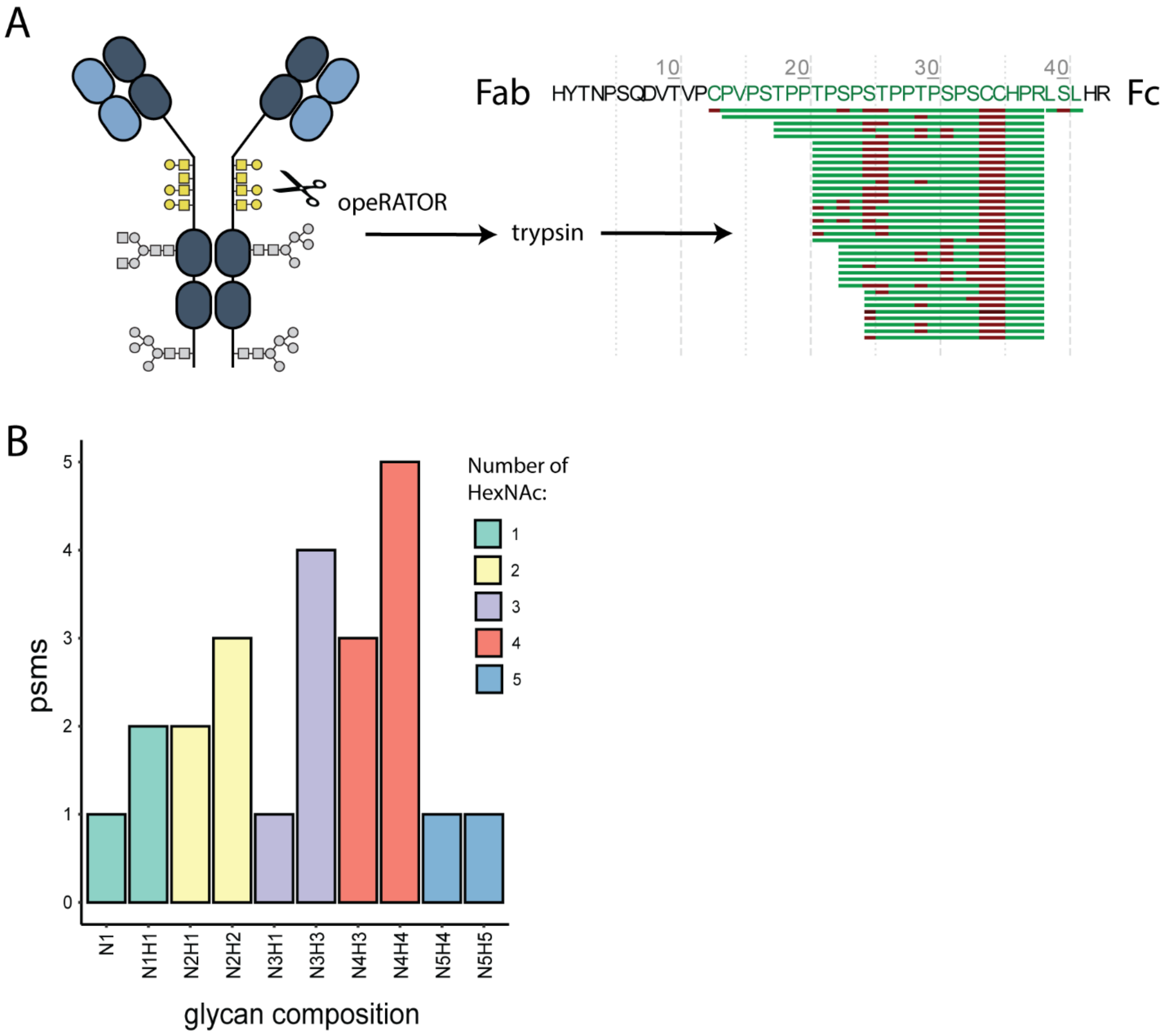

Figure S3. *O*-glycoproteomics analysis of the anti-CD20 IgA1 after digestion with OpeRATOR and trypsin. A) Peptide coverage map for the peptides in the hinge region, where green is unmodified amino acid residue and red is modified amino acid residue. The modification on the C is carbamidomethyl, and modifications on S/T are *O*-glycans. This data revealed that OpeRATOR can cleave the IgA1 hinge region at different sites and that multiple S/T sites on the Fc end of the hinge remain occupied by *O*-glycans. B) Number of PSMs identified bearing the glycan composition on the x-axis, where N is HexNAc and H is Hex. PSMs were filtered for a Byonic Score higher than 150.

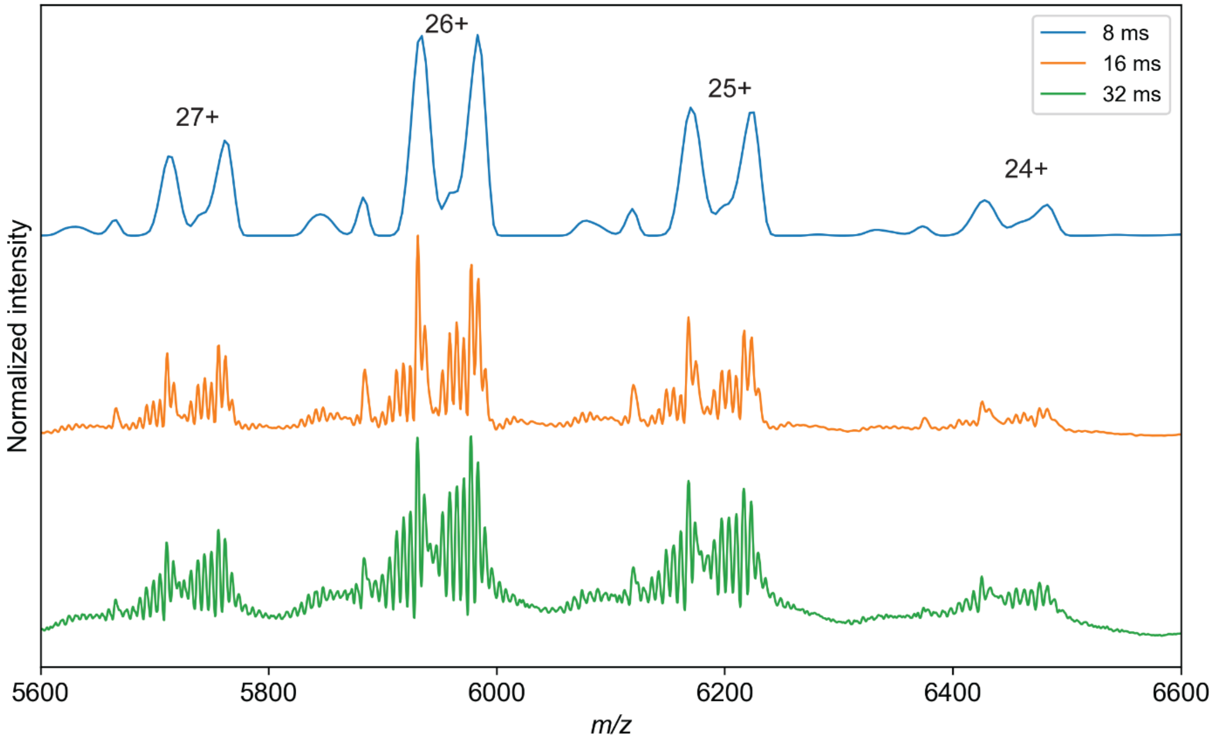

Figure S4. Native mass spectrometry of recombinant anti-CD20 IgA1 after incubation with OpeRATOR harboring just a limited set of residual glycoproteoforms. Degrading a major part of the glycoproteoforms on intact IgA1, leads to simplified native MS spectra that become better resolved as the transient time is increased. Here is shown the 5600-6600 *m/z* window. The corresponding full MS spectrum is shown in Figure 3A. As described in the main text, each charge state reveals three cluster of peaks, corresponding to the occupancies of 2, 3 and 4 N-glycan sites, respectively.
